# Burden and risk factors for snakebite in India: protocol for a systematic review

**DOI:** 10.12688/f1000research.21924.2

**Published:** 2020-04-02

**Authors:** Soumyadeep Bhaumik, Robyn Norton, Jagnoor Jagnoor

**Affiliations:** 1 The George Institute for Global Health, University of New South Wales, Sydney, New South Wales, Australia; 2 The George Institute for Global Health, New Delhi, India; 3 The George Institute for Global Health, Nuffield Department of Women's and Reproductive Health, University of Oxford, Oxford, UK

**Keywords:** Snake Bites, Epidemiology, India, Prevalence, Incidence, risk factor, Health systems, economic costs

## Abstract

**Introduction: **Snakebite is a neglected tropical disease with a high burden in South and South-East Asia and sub-Saharan Africa. In 2019, the World Health Organization (WHO) released a roadmap which aims for a 50% reduction in death and disability due to snakebite globally by 2030. It is estimated that India has the highest number of snakebite deaths in the world.

**Objective: **To synthesize evidence on the burden (incidence/ prevalence, mortality, morbidity, health facility and economic), and risk factors for snakebite in India.

**Methods: **We will search for peer-reviewed literature and grey literature in six electronic databases (MEDLINE, EMBASE, Global Health, PsychInfo, CENTRAL, SafetyLit) and hand-search IndMed, conference abstracts, relevant websites and citation tracking. Two reviewers will screen and extract data independently with a third reviewer acting as an arbiter for any inconsistencies. Quality of the included studies will be assessed using the Joanna Briggs Institute (JBI) critical appraisal tools.

For burden, data from facility based and community-based studies will be synthesised and reported separately, except for studies conducted concurrently. We will conduct meta-analysis for community-based studies at state-level for incidence/prevalence, mortality and morbidity , if appropriate. The PROGRESS Plus lens will be used to explore equity .

Analyses for each individual risk factor-outcome pair will be conducted and reported separately. If appropriate, meta-analyses will be conducted as per JBI guidelines, assessing heterogeneity using Tau-squared, Cochran’s Q test and Chi-squared (p > 0.05) tests. We plan to conduct sub-group analyses based on pre-specific parameters. A funnel plot will be generated if there are more than nine studies included in a specific meta-analysis, to assess publication bias

When meta-analysis is not appropriate, structured tabulation of results across studies and/or by vote counting based on the direction of effect as per guidelines in the Cochrane Handbook.

## Background

Snakebite is a neglected tropical disease, with considerable burden in South Asia, Southeast Asia, and sub-Saharan Africa ^1^. They are known to affect rural, indigenous and economically disempowered communities who lack political voice ^2– 4^. A modelling study using data on venomous snake distribution, health-care access, and availability of snake anti-venom, estimated that globally 146.70 million people live in snakebite prone areas lacking quality health-care provisions ^5^. However, broad consensus is that these numbers are underestimates as many affected by snakebite are ‘out-of-reach’ of the formal health systems ^6, 7^. Snakebite envenomation also causes long-term health effects, and is believed to have high social and economic impacts in affected communities ^6, 8, 9^. Morbidity and socio-economic impact of snakebite is not well understood and remains under-researched globally ^9, 10^.

In 2018, recognising the public health impact of snakebite on vulnerable communities the World Health Assembly (WHA) passed a resolution to address the burden of snakebite ^4^. Earlier in 2019 the World Health Organization (WHO) released a roadmap which aims to halve by 2030 the death and disability due to snakebite globally ^11^. The WHO strategy rests on four pillars of action: empowering and engaging communities; ensuring safe, effective treatment; strengthening health systems; and increasing partnerships, coordination and resource usage through collaborations ^11^.

More than a third of the global deaths, about 46,000 annually, are estimated to occur in India ^12^ with not much known about other aspects of burden ^7^ or risk factors in the country. Understanding the epidemiology of snakebites (in terms of incidence/prevalence of bites and envenoming, mortality, morbidity and risk factors) at the national and subnational level together with economic costs and health facility burden is critical for developing strategies, plans and programs to address the burden of snakebite. There are no systematic reviews on the burden and risk factors for snakebite in India, although evidence synthesis on burden and impact has been done for other countries or regions ^13– 16^. The current article provides the protocol for a systematic review on the burden and risk factors for snakebite in India.

## Objectives

To synthesize evidence on the burden (incidence/prevalence, mortality, morbidity, health facility and economic), and risk factors for snakebite in India

## Research questions

1. What is the burden (incidence/prevalence, mortality, morbidity, health facility, and economic) of snakebite in India nationally and sub-nationally?
2. What are the risk factors related to snakebite (bite and death) in India?

## Protocol and registration

The objectives, inclusion criteria and methods of analysis for this systematic review are specified in advance and documented in this *a priori* protocol.

## Eligibility criteria

The systematic review consists of two distinct evidence syntheses - burden (incidence/prevalence, mortality, morbidity, health facility and economic); and risk factors for snakebite (bite and death). Synthesis of evidence for each domain will be conducted and reported separately in alignment with recent Cochrane guidelines ^17^.

### Eligibility criteria for evidence synthesis on the burden of snakebite in India

We will include studies that meet all the following criteria:

• **Population** – involving human participants from India, irrespective of age, gender or any other characteristics.
• **Condition** – snakebite irrespective of how it is diagnosed, measured or confirmed.
• **Setting -** facility or community-based studies; autopsy-based studies will be included for understanding aspects of burden, as relevant.
• **Burden Outcomes-** studies reporting any of the following outcomes will be included –
  ○ Incidence/prevalence– incidence rate of snakebite or snakebite envenoming (i.e. clinical envenoming) (population or age-specific) from community-based studies only; prevalence rate of snakebite or snakebite envenoming from community-based, autopsy-based and facility-based studies;
  ○ Mortality – incidence death rate (mortality rates per 100,000) due to snakebite (population or age-specific) from community-based studies only; case fatality rate due to snakebite from facility-based studies.
  ○ Morbidity – measured using any validated disability or quality of life tools or DALYs or any other standardised measure (as defined by the authors) from community and facility-based studies.
  ○ Health facility burden- measured in terms of proportions and/or percentages for any of the following outcomes (from facility-based studies only):
    ▪ Visits/admissions in emergency department, clinic/out-patient department, in-patient department (for both venomous and non-venomous bites)
    ▪ Days of inpatient admission (for both venomous and non-venomous bites)
    ▪ Requirement of specialist consultation
    ▪ Requirement for referral in higher facility
    ▪ Requirement of ventilatory support / dialysis support / blood transfusion in acute setting (as defined by primary study authors)
    ▪ Requirement of fasciotomy to manage compartment syndrome (as defined by primary study authors)
    ▪ Requirement of long-term rehabilitation support (as defined by primary study authors)
  ○ Economic burden- from provider perspective or client perspective (direct and/or indirect costs) – as defined and measured by primary study authors (from community-based or facility-based studies).
  ○ **Study design –**
  ○ cohort studies (prospective or retrospective), or
  ○ cross-sectional studies (analytical)
  • There will be no restriction by year of publication or language.

### Eligibility criteria for evidence synthesis of risk factors for snakebite in India

We will include studies that meet all the following criteria:

- **Population** – involving human participants with snakebite or at-risk of snakebite from India. We will not include forensic- autopsy studies for understanding risk factors.
- **Setting:** facility or community-based studies; autopsy-based studies will be excluded as they cannot give data on risk factors.
- **Risk factors of interest and related outcomes -**No *a priori* list of risk factors is listed as the scope of the evidence synthesis is broad. We will include any risk factor related to following outcomes:
  ○ incidence of snakebite or death due to snakebite from community-based studies (reported in terms of relative risks (RR), odds ratios (OR), hazard ratios (HR), standardized incidence ratios (SIR) or a standardized mortality ratios (SMR); adjusted or otherwise)
  ○ death due to snakebite (case fatality) from facility-based studies (reported in terms of relative risks (RR), odds ratios (OR), hazard ratios (HR), standardized incidence ratios (SIR) or a standardized mortality ratios (SMR); adjusted or otherwise)

- **Study design –**We will include the following study designs:
  ○ cohort studies (prospective and retrospective), or
  ○ case-control studies, or
  ○ cross-sectional studies (analytical)

We will not include risk-modelling studies as they are not within the scope of the current evidence synthesis.

- There will be no restriction by year of publication or language.

## Information sources and search strategy

### Electronic databases

We will search the following electronic databases for eligible studies using adaptions of the MEDLINE search strategy developed for this purpose (see extended data ^18^):

- MEDLINE
- EMBASE
- Global Health
- PsychInfo
- CENTRAL
- SafetyLit

### Searching other resources

We will hand-search IndMed (a bibliographic database covering prominent peer reviewed Indian biomedical journals), conference abstracts (including but not limited to Indian Public Health Association Conference - IPHACON, Annual Conference of the Toxinological Society of India- TSICON, Annual National Conference of Indian Society Of Toxicology - TOXOCON: as available) and contact researchers of repute in India to identify more studies. We will also hand search vital statistics data, government reports, population surveys or white papers which have reported on the burden and/or risk factors for snakebite specifically in relevant websites. We will also hand search the reference lists of all included studies found by other methods to retrieve additional records.

## Study selection

Two review authors will independently assess the eligibility of primary studies based on titles and/or abstracts in the first phase. We will then acquire the full text of all papers identified as potentially relevant by at least one review author. Two review authors will then assess these papers independently and classify them into four categories – included for burden; included for risk factors; included for both burden and risk factors; excluded. We will resolve disagreements, by discussion with a third reviewer acting as an arbiter. We will attempt to contact study authors for further information, if necessary.

## Data management

We will extract data using a standardised data extraction protocol, developed by adding extra data elements to the JBI recommended minimum standards for data extraction for prevalence, incidence and risk factor systematic reviews ^19, 20^. This will be done by piloting the tool (independently and then reaching a consensus) on five studies (each from burden and risk factors) chosen randomly from the list of included studies. Data management will be done using the Joanna Briggs Institute- The System for the Unified Management, Assessment and Review of Information (SUMARI).

## Quality of included studies

We will appraise the quality of the included studies by using the JBI quality assessment tools for cohort, analytical cross-sectional and case-control studies ^19, 20^.

## Synthesis of results

### Synthesis methods for evidence synthesis on the burden of snakebite in India

Data from facility based and community-based studies will be synthesised and reported separately, except in the case of studies which have conducted both concurrently.

An equity lens will be applied to understand burden in a granular fashion. We will use the PROGRESS plus framework ^21^ for this purpose and extract and synthesise disaggregated data, if available on the framework parameters (PROGRESS-Plus - Place of residence; Race/tribal people; Occupation; Gender/sex; Religion; Education; Socioeconomic status; Social capital; and “Plus” to indicate other possible equity factors which might affect the outcomes of interest in relation to snakebite).

### For incidence/prevalence, mortality and morbidity outcomes

Snakebite as a condition is known to be localised in nature. As such, pooling of data from heterogenous studies into one pooled national-level estimate will not reflect the variability in the burden of the condition at sub-national and local levels. The phenomenon of diluting the burden of snakebite by pooling of specific local data into national snakebite incident rate data has been previously recognised and been described as the ‘tyranny of mean values’ ^6^. As such we will not pool data to conduct meta-analysis at the national level. 

We will conduct meta-analysis by pooling data from community -based studies at the state level using current political boundaries ( Political Map of India, 9 ^th^ Edition, 2019, Surveyor General of India) for incidence/prevalence, mortality and morbidity. We will not pool data from any facility-based studies, as incidence/prevalence, mortality and morbidity from them will be dependent on patient, health facility and catchment area characteristics implying considerable clinical heterogeneity. 

We will use the random effects model with 95% CI for incidence/prevalence , mortality and morbidity as per JBI guidelines since the assumption of one true effect for a fixed model is usually not true for prevalence and incidence data ^19^. We will use a fixed-effect model with 95% CI only if we assess heterogeneity (clinical, methodological or statistical) to be significant. Statistical heterogeneity will be considered significant only if it is >40%. Heterogeneity will be assessed by Tau-squared, Cochran’s Q test and Chi-squared (p > 0.05) tests ^19^. 

If meta-analysis is not appropriate, we will summarise estimates using a structured tabulation of results across studies (arranged chronologically) to report range and magnitude of observed values and/or by vote counting based on the direction of effect as per guidelines in the Cochrane Handbook ^22^. Results from vote counting will be reported alongside any available individual estimates study using associated visualisation like harvest plot or effect direction plot, as appropriate ^22^.

We plan to conduct sub-group analyses for the following, if enough studies are found:

- Sex/gender (male; female; other)
- Age groups: Children (less than 10 years), adolescent (11–19 years), young adults (20–24 years)
- Tribal / non-tribal people
- Occupation (agricultural/plantation workers or farmers, and fishermen)
- Any other PROGRESS-Plus characteristics

Sensitivity analyses will be conducted, as appropriate, and if enough studies are available, to assess robustness of results (based on assessment of different risk of bias parameters and sample size). Additional sensitivity analysis other than what is mentioned *a priori* might be conducted. We will generate a funnel plot to assess publication bias if there are more than nine studies included in a specific meta-analysis. Funnel plot asymmetry will be tested by statistical tests (Egger test, Begg test, Harbord test) as appropriate.

### For health facility burden and economic burden outcomes

- We will not conduct meta-analysis for health facility burden and economic burden as the same is inappropriate because of heterogeneity across different health facilities owing to differences in characteristics of catchment areas, the facility itself and patient characteristics. We will summarise effect estimates using a structured tabulation of results across studies (arranged state wise) to report range and magnitude of observed values as per guidelines in the Cochrane Handbook ^22^. If enough studies are available we will report for different sub-groups based on: Type of health facility (government; private; non-profit)
- Level of health facility (primary health centre; community health centre; sub-divisional or district hospital)
- for economic burden - income levels (income quartiles or any other as defined by study authors ) or any other PROGRESS-Plus characteristics

### Synthesis methods for evidence synthesis of risk factors for snakebite in India

Analysis for each individual risk-factor outcome pair will be conducted and reported separately. We will use the random effects model with 95% CI as per JBI guidelines. We will use a fixed-effect approach only if we assess heterogeneity (clinical, methodological or statistical) to be significant. Statistical heterogeneity will be considered significant only if it is >40%. Heterogeneity will be assessed by Tau-squared, Cochran’s Q test and Chi-squared (p > 0.05) tests ^20^.

If meta-analysis is not appropriate, we will summarise estimates using a structured tabulation of results across studies (arranged risk-factor wise)to report range and magnitude of observed values and/or by vote counting based on the direction of effect as per guidelines in the Cochrane Handbook ^22^. Results from vote counting will be reported alongside any available individual estimates study using associated visualisation like harvest plot or effect direction plot, as appropriate ^22^.

We plan to conduct sub-group analyses for the following, if enough studies are found based on:

- Study design
- Setting (community based; facility based)
- Sex/gender (male; female; other)
- Age groups: Children (less than 10 years), adolescent (11–19 years), young adults (20–24 years)
- Tribal / non-tribal people
- Occupation (agricultural/plantation workers or farmers, and fishermen)
- Any other PROGRESS-Plus characteristics

Sensitivity analyses will be conducted, as appropriate, and if enough studies are available, to assess robustness of results (based on assessment of different risk of bias parameters and sample size). Additional sensitivity analysis other than what is mentioned *a priori* might be conducted. We will generate a funnel plot to assess publication bias if there are more than nine studies included in a specific meta-analysis. Funnel plot asymmetry will be tested by statistical tests (Egger test, Begg test, Harbord test) as appropriate.

### Dissemination of information

We will publish the results of this review and will make the data accessible openly in re-usable format. The data will also be disseminated through evidence summaries and policy briefs to stakeholders in governments, public institutions and communities.

### Study status

The search, screening and subsequent steps will be undertaken after the protocol completes peer-review.

## Data availability

### Underlying data

All data underlying the results are available as part of the article and no additional source data are required.

### Extended data

Figshare: Extended Data Set : Burden and risk factors for snakebite in India: protocol for a systematic review. https://doi.org/10.6084/m9.figshare.11536776.v2 ^18^ ****

This project contains the following extended data:

- Prisma-P Checklist
- Study search strategy

### Reporting guidelines

PRISMA-P checklist for ‘Burden and risk factors for snakebite in India: protocol for a systematic review’. https://doi.org/10.6084/m9.figshare.11536776.v2 ^18^

Data are available under the terms of the Creative Commons Attribution 4.0 International license (CC-BY 4.0).
