# Supplementary material for: Burden and risk factors for snakebite in India: protocol for a systematic review
Source: F1000Res. 2020 Apr 2;9:25. Originally published 2020 Jan 16. [Version 2] doi: 10.12688/f1000research.21924.2 (PMC7194469; doi:10.12688/f1000research.21924.2)
Supplement: Peer review history

# Peer review: Reviewer response for version 2

**DOI:** 10.5256/f1000research.25296.r61923

**Published:** 2020-04-14

**Authors:** Abul M. Faiz

**Affiliations:** 1 Dev Care Foundation, Dhaka, Bangladesh

The authors revised the article carefully and collected different studies using cutting edge methodology. Though, we are a bit confused for repetition of word in study design mentioned on page no-4. Same on page 6 repetition of subgroup analysis, otherwise the paper is well written and structured. However, in our opinion the paper has some shortcomings with regard to some text but it may be accepted.

Is the study design appropriate for the research question?

Yes

Is the rationale for, and objectives of, the study clearly described?

Yes

Are sufficient details of the methods provided to allow replication by others?

Partly

Are the datasets clearly presented in a useable and accessible format?

Yes

Reviewer Expertise:

NA

I confirm that I have read this submission and believe that I have an appropriate level of expertise to confirm that it is of an acceptable scientific standard.

# Peer review: Reviewer response for version 2

**DOI:** 10.5256/f1000research.25296.r61924

**Published:** 2020-04-09

**Authors:** Bert Avau

**Affiliations:** 1 Centre for Evidence-Based Practice (CEBaP), Belgian Red Cross, Mechelen, Belgium; 2 Cochrane Belgium, Leuven, Belgium

No further comments.

Is the study design appropriate for the research question?

Yes

Is the rationale for, and objectives of, the study clearly described?

Yes

Are sufficient details of the methods provided to allow replication by others?

Partly

Are the datasets clearly presented in a useable and accessible format?

Yes

Reviewer Expertise:

Evidence synthesis methodology

I confirm that I have read this submission and believe that I have an appropriate level of expertise to confirm that it is of an acceptable scientific standard.

# Peer review: Reviewer response for version 1

**DOI:** 10.5256/f1000research.24174.r58690

**Published:** 2020-02-17

**Authors:** Abul M. Faiz, Ariful Bashar

**Affiliations:** 1 Dev Care Foundation, Dhaka, Bangladesh; 2 Bangabandhu Sheikh Mujib Medical University (BSSMU), Dhaka, Bangladesh

The objective related to Burden and risk factors are clumped with incidence/prevalence, mortality and morbidity and envenomation; may be provided in more elaboration specially risk factor because adverse outcome and death is not risk factor for envenomation. The authors have reported their work appropriately according to the PRISMA-P checklist.

 Eligibility criteria: It should be made clear that all or part of the eligibility criteria will need to be fulfilled. Risk factor is not properly addressed here and authors has no plan for risk modelling. Risk variables are not clear too. I think more emphasis was given on burden. So more clarification and clear objective about risk factor should be mentioned.

 Condition: ‘irrespective of how it was diagnosed’- is it, that venomous and non venomous bites will be grouped together which will cause problem in interpretation of the results.

 Dataset search strategy: It can also include venomous and antivenom as we consider economic burden .

 Synthesis of result: It is not unusual to get heterogeneous result due to regional variability and low impact article. So how the authors analyse this heterogeneity should be mentioned.

 Health facility burden (page 3): Usually all/most of the patients of snakebite present at the emergency department unless manifested as chronic condition. Definition/clarity is required on ‘specialist’, ‘higher facility’, ‘compartmental syndrome’, ‘long term rehabilitation support’. How uniformity among the investigators on decision for ventilatory support, dialysis, blood transfusion will be interpreted. How the individual component of data will be collected.

 Study design (page 4): It would not be wise to have a study having no restriction of ‘year of publication’ to see the burden and risk factors which may change over time.

 Risk factors: a list may be developed. 2 ^nd^ bullet is not clear- snakebite irrespective of envenomation may also be admitted, so admission does not necessarily mean envenomation.

 Searching other sources: how the authors will identify ‘researchers of repute’.

Is the study design appropriate for the research question?

Yes

Is the rationale for, and objectives of, the study clearly described?

Yes

Are sufficient details of the methods provided to allow replication by others?

Partly

Are the datasets clearly presented in a useable and accessible format?

Yes

Reviewer Expertise:

Clinician and Toxicologist

We confirm that we have read this submission and believe that we have an appropriate level of expertise to confirm that it is of an acceptable scientific standard, however we have significant reservations, as outlined above.

# Response

**Published:** 2020-03-17

**Authors:** Soumyadeep Bhaumik

**Affiliations:** PHFI, India

We thank the reviewer for the very useful comments and would like to clarify that we already have two separate review questions and corresponding sections. We elaborate concerns below :

- Burden is conceptualised as incidence/prevalence, mortality, morbidity, health facility and economic
- Risk factors- We agree with the concern about the risk factor and clarify and in lieu of reviewer feedback have revised to include the following two only and removed adverse outcome and envenomation as suggested: 
  - Risk factor for a snakebite- this contributed to preventive aspects of bite itself 
  - Risk factor for death due to snakebite

Based on the comments, we have reworded the section to provide more clarity. 

 Eligibility criteria: We have revised the manuscript, to provide more clarity on the eligibility criteria.

 Risk factor: We have clarified the objectives for the risk factors as above.  We clarify that we do not intend to or plan to do risk modelling as this is a systematic review and not a risk modelling study. Risk modelling cannot be done with summary statistics available form published literature.  We have detailed methods for meta-analysis.

 Condition: We thank the reviewer for the comment. We have considered this already and since we are conducting a systematic review of published studies, we must use diagnostic definitions as used by primary study authors. Hence the use of irrespective. Primary study authors would classify venomous and non-venomous and their definitions will be used. 

 Dataset search strategy : We have revised the search strategy to include key words related to snake anti-venom as per reviewer comments. The keywords added in search are :

 “anti-venom* or antivenom* or anti-dote* or antidote* or anti-snake or antisnake”

 We have not included venomous in the search strategy as it leads to decrease in the sensitivity of the search leading to retrieval of many studies on venomous animals including snake . These are not on snakebites. Any study which has looked at snakebite burden and risk factor will have the word “bite” and will be retrieved from this current search strategy.

 The changes are reflected in the dataset.

 Synthesis of result: We have now elaborated on this section to clarify that for the purpose of the review will be consider > 40% heterogeneity to be heterogenous

 Health facility burden : We agree to the observation that most bites present at emergency department but we do not want to be exclude those who visit clinic/out-patient department, in-patient department, so as to comprehensively report on health system burden, inclusive of for disability/chronic condition resulting from snake bite.

 Definition/clarity is required on ‘specialist’, ‘higher facility’, ‘compartmental syndrome’, ‘long term rehabilitation support’:  We thank the reviewer but since it is a systematic review of already conducted primary studies, we are going to use the definition as per primary study authors. We cannot control how individual component of data is collected- this may or may not be uniform, but we will collect and report the different definitions /modalities of measurement along with the outcome data.

 We chose to put no time restrictions, so as to report change over time.

 Risk factors : We have not made an a priori list as we are looking to review all risk factors. This is also not required as this is not a primary study and out search strategy has been made broad to look for all studies on snakebite in India.

 We have included admission as part of burden. We conceptualised this as health system burden since both venomous and non-venomous bites need evaluation in a health facility and is hence contributing to health system burden. As mentioned, we are collecting this data for both venomous and non-venomous bites and these will be reported separately.

 Searching other sources: We will contact experts as per knowledge of the review team- this is a standard evidence synthesis approach. Detailed number of experts who contributed to identification of newer studies will be provided at full review phase.

# Peer review: Reviewer response for version 1

**DOI:** 10.5256/f1000research.24174.r59189

**Published:** 2020-02-04

**Authors:** Bert Avau

**Affiliations:** 1 Centre for Evidence-Based Practice (CEBaP), Belgian Red Cross, Mechelen, Belgium; 2 Cochrane Belgium, Leuven, Belgium

The authors of this systematic review protocol aim to map the burden and risk factors for snakebite in the Indian context. To do so, they have composed two separate review questions: one on the incidence and prevalence of snakebite, the other one on the risk factors that may contribute to the burden of snakebite.

 A systematic review of the existing literature seems an appropriate method to reach their objectives.

 The authors have reported their work appropriately according to the PRISMA-P checklist. However, several suggestions for improving/clarifying the reporting of their work are made below, especially regarding synthesizing their findings.

 The authors include as datasets a completed PRISMA-P checklist and a search string to search for paper in Ovid Medline. These datasets are in my opinion accessible and useable.

 Specific points for consideration regarding the reporting of the methods provided:

 1) Page 4 - Eligibility criteria for risk factors for snakebite in India:Can you please elaborate as to why risk modelling studies are not within scope of this systematic review? The scope of this review is as I understood broad, summarizing all the evidence on burden and risk factors for snakebite. To my understanding, risk modelling studies may contribute to the scope as set by the authors.

 2) Dataset search strategy:

 I would search for "envenom*" instead of "envenomation*". This will make sure you also find records that use the term "snake envenoming" instead of "snake envenomation".

 3) Page 5 - Synthesis methods for evidence synthesis on the burden of snakebite in India:

 Though I agree it may be misleading to pool data from different diverse regions into one national summary, it may be worthwhile to consider whether combining findings from studies from the same (or similar) regions is possible. Therefore I’m not sure whether a priori excluding the possibility of a meta-analysis for incidence/prevalence data is the best option here.

 4) Page 5 - Synthesis methods for evidence synthesis of risk factors for snakebite in India:

 It is generally not advisable to choose the type of meta-analysis method (fixed or random) a posteriori, based on the observed level of heterogeneity (JBI Handbook section 5.5.8.2 & Cochrane Handbook section 10.10.4.1 ^1^). Rather, one should consider the underlying assumptions of the two models and decide a priori which method would likely be the most appropriate in relation to the expected type of data. Please address this issue.

 5) Page 5 -  Synthesis of results:

 In case meta-analyses are considered inappropriate, one will need to synthesize the data in another way. Please provide a method for doing this. The guidance by Campbell et al. ^2^ and Chapter 12 of the Cochrane Handbook ^3^ may provide valuable input for this.

 6) Page 5 - Synthesis of results:

 In case a meta-analysis can be performed, it is not unlikely that you will encounter statistically heterogeneous results. Please elaborate on how you will assess heterogeneity (i.e. what will you consider a heterogenous result?). Guidance on this can be found in Chapter 10.10 of the Cochrane Handbook ^4^.

 7) PRISMA-P Item 12:

 Although the authors state “a standardised data extraction protocol, developed by adding extra data elements to the JBI recommended minimum standards for data extraction for prevalence, incidence and risk factor systematic reviews” will be used, it is not clear which items the authors will extract. It would be transparent to add an empty copy of this data extraction sheet, to be clear about which data items will be extracted.

 8) PRISMA-P Item 14:

 The authors need to clarify how they will use the risk of bias assessment during data synthesis.

 9)  PRISMA-P Item 17:

 I disagree that assessment of the body of evidence is not applicable. Several studies will contribute to outcomes, therefore it is useful to assess our overall confidence in the evidence gathered from different studies. I’m not saying the authors should definitely use the GRADE approach for this, but being transparent in how the overall strength of the body of evidence will be evaluated is necessary.

Is the study design appropriate for the research question?

Yes

Is the rationale for, and objectives of, the study clearly described?

Yes

Are sufficient details of the methods provided to allow replication by others?

Partly

Are the datasets clearly presented in a useable and accessible format?

Yes

Reviewer Expertise:

Evidence synthesis methodology

I confirm that I have read this submission and believe that I have an appropriate level of expertise to confirm that it is of an acceptable scientific standard, however I have significant reservations, as outlined above.

## References

[1] : Fixed and random-effects estimates.2019; Reference source

[2] : Synthesis without meta-analysis (SWiM) in systematic reviews: reporting guideline. *BMJ*.2020; 10.1136/bmj.l6890 10.1136/bmj.l6890 PMC719026631948937

[3] : Chapter 12: Synthesizing and presenting findings using other methods. Reference source

[4] : What is heterogeneity?. Reference source

# Response

**Published:** 2020-03-17

**Authors:** Soumyadeep Bhaumik

**Affiliations:** PHFI, India

We thank the reviewer for the comments on the scope and appropriate methods as well as the suggestions for improvement and clarification has been useful to us for revising the protocol (numbers in response correspond to numbers in query . 

 1.    We thank the reviewer for consideration of inclusion of risk modelling studies.  We have followed the guidelines of JBI (Joanna Briggs Institute) for systematic reviews of risk. The guidelines recommend study designs and we have included them : https://wiki.joannabriggs.org/display/MANUAL/7.2.1+Observational+Study+Designs  .  Risk modelling studies are not recommended study design for inclusion. 

 A risk modelling study is a statistical procedure for assigning a probability of developing a future outcome.  We contend that this is not within the purview of this evidence synthesis as risk modelling studies look into prediction in contrast to cohort or case-control studies. 

 2.    We have now modified the search strategy in the dataset.  

 3.    Thank you for acknowledging that a national pooling might be misleading. We have had considerable discussion on the issue within the team and now looked at the issue of burden being meta-analysed more granularly and made substantial changes to reflect this in the manuscript. The summary of the new analysis for synthesising evidence is provided: 

 •    We conceptualised burden to go beyond incidence or mortality, to mean the following which are reflected as outcomes: incidence/prevalence, mortality, morbidity, health facility and economic. Data from these come from either community based or facility based or autopsy-based studies. 

 •    For incidence/prevalence, mortality and morbidity we will pool data at state level and conduct meta-analysis (if appropriate) in a state-wise fashion (political borders as per Surveyor General of India – May 2020) from community-based studies only. We will not pool data from any facility based studies as data from them will be dependent on patient , health  facility and catchment area characteristics implying considerable clinical heterogeneity 

 •    We will not pool data from health facility and economic outcomes and conduct meta-analysis for the same reasons as above. We have provided more information on what will be done when meta-analysis is not envisaged subsequently.

 4.    We have now elaborated on the section to reflect changes in alignment with suggestion. In summary, we have amended to align with the JBI recommendation of using the random effects model a priori. We will use a fixed-effect approach only if we assess heterogeneity (clinical and methodological and statistical) to be minimal.

 5.    We have earlier mentioned on Page 5 that we will assess patterns in the data through tabulation of results based on JBI guidelines. We have now taken note of the new Cochrane Handbook and added additional information on analysis methods where meta-analysis is not possible or not envisaged .It is revised to now include information about the structured reporting of results guidelines provided by the reviewer and following approaches will be taken as appropriate for different outcomes. A summary is provide below  : 

 •    Summarizing effect estimates to report range and distribution of observed values 

 •    Vote counting based on direction of effect (with on comment on the magnitude of effect) using harvest plot and/or effect direction plot.

 •    We will use visualisation tools,  as appropriate (harvest plot, effect direction plot, etc).

 We have also elaborated on sub-group analysis being done for the same as relevant for different analysis.

 6.    We have now elaborated on this section to clarify that for the purpose of the review will be consider > 40% heterogeneity to be a “heterogenous result” and have detailed methods used for the same 

 7.    We have further elaborated to mention that the data extraction form will be pilot tested in the first few studies. Providing an empty data extraction form is not required as per PRISMA-P guidelines. We have now put information on the “planned method of extracting data from reports (such as piloting forms, done independently, in duplicate), any processes for obtaining and confirming data from investigators” as required in PRISMA-P. We reiterate and as noted in out transparency statement, all data will be provided with the systematic review findings.

 8.    We have clarified further on the use of sensitivity analysis if meta-analysis and how the use the risk of bias assessments will be used during the data synthesis process. 

 9.    We agree that using GRADE to assess overall confidence in evidence would be useful, however GRADE is not an appropriate tool for evidence synthesis for burden and risk factors. GRADE is suitable for interventions and diagnostic test accuracy studies only and GRADECerQUAL for qualitative studies. To the best of our knowledge there are no appropriate approach available for our purpose, for which consensus tool exist.    

 PRISMA-P is a reporting guideline and not a methodological guidance and we have reported that overall strength of body of evidence will not be assessed in our research and rendering corresponding section in PRISMA-P not applicable.
